# Supplementary material for: Are nitrogen and carbon cycle processes impacted by common stream antibiotics? A comparative assessment of single vs. mixture exposures
Source: PLoS One. 2022 Jan 5;17(1):e0261714. doi: 10.1371/journal.pone.0261714 (PMC8730405; doi:10.1371/journal.pone.0261714)
Supplement: S1 File — Sediment particle size composition of stream sediment. (PDF) [file pone.0261714.s001.pdf]

S1.
Sediment particle size composition of stream sediment

Classification	Size Range	% composition
Size class 1	≥ 1mm	14.6
Size class 2	425 μm to 1 mm	39.5
Size class 3	250 μm to 425 μm	31.4
Size class 4	106 μm to 250 μm	13.2
Size class 5	< 106 μm	0.67
