# Supplementary figures and images for: Are nitrogen and carbon cycle processes impacted by common stream antibiotics? A comparative assessment of single vs. mixture exposures
Source: PLoS One. 2022 Jan 5;17(1):e0261714. doi: 10.1371/journal.pone.0261714 (PMC8730405; doi:10.1371/journal.pone.0261714)

S2.

Sediment oxygen levels from ambient and nutrient enrichment

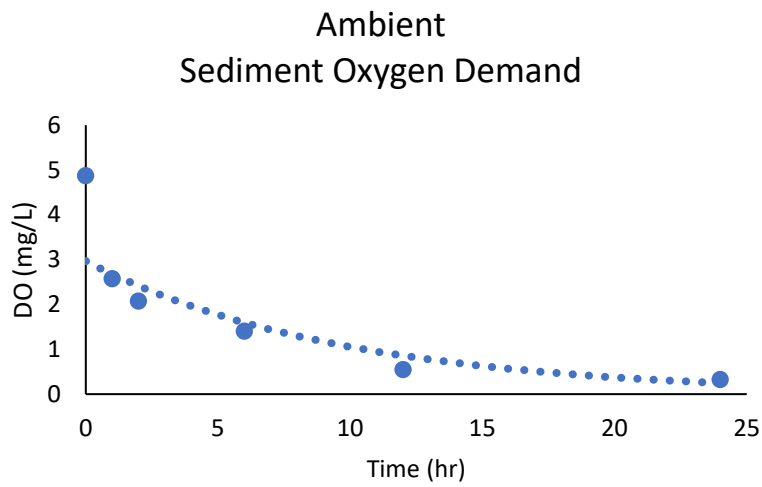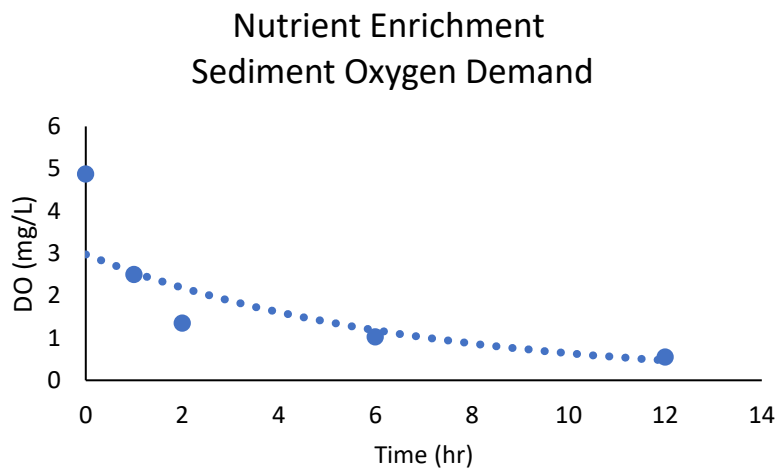

Supplement: S2 File — Sediment oxygen levels from ambient and nutrient enrichment. (PDF) [file pone.0261714.s002.pdf]
