# Supplementary material for: Are nitrogen and carbon cycle processes impacted by common stream antibiotics? A comparative assessment of single vs. mixture exposures
Source: PLoS One. 2022 Jan 5;17(1):e0261714. doi: 10.1371/journal.pone.0261714 (PMC8730405; doi:10.1371/journal.pone.0261714)
Supplement: S3 File — Mean ± SE NH4+ and NO3- concentrations at each sampling point. (PDF) [file pone.0261714.s003.pdf]

S3.

Mean ± SE NH_4^+ and NO_3^- concentrations at each sampling point

	NH_4^+ (µg N L^{-1})			
	**Hour 0**	**Hour 6**	**Hour 12**	**Hour 24**
Control	459 ± 46	519 ± 41	448 ± 115	33 ± 9.4
SMX	388 ± 9.8	436 ± 20	532 ± 49	391 ± 39
DAN	NA	460 ± 15	528± 49	259 ± 139
ETM	673 ± 49	470 ± 40	531 ± 51	186 ± 44
Mixture	493 ± 5.8	565 ± 12	784 ± 24	754 ± 9.5
	NO_3^-(µg N L^{-1})			
	**Hour 0**	**Hour 6**	**Hour 12**	**Hour 24**
Control	166 ± 9	208 ± 35	123 ± 20	29 ± 10
SMX	281 ± 6.9	233 ± 13	175 ± 4.2	126 ± 5.9
DAN	269 ± 9	245 ± 15	166 ± 5.2	126 ± 2.3
ETM	269 ± 4	240 ± 4.3	190 ± 13.1	120 ± 5.7
Mixture	233 ± 0.5	233 ± 9	167 ± 3	125 ± 15.3

NA= non-applicable, DAN NH_4^+ data was not included at 0 h due to statistical outliers. L^{-1}= liters, N= nitrogen
