# Supplementary material for: Are nitrogen and carbon cycle processes impacted by common stream antibiotics? A comparative assessment of single vs. mixture exposures
Source: PLoS One. 2022 Jan 5;17(1):e0261714. doi: 10.1371/journal.pone.0261714 (PMC8730405; doi:10.1371/journal.pone.0261714)
Supplement: S4 File — Mean ± SE concentrations of N2O, N2, CH4, and CO2 from each experimental treatment over the 7-day study. (PDF) [file pone.0261714.s004.pdf]

S4.
Mean ± SE concentrations of N_2O, N_2, CH_4, and CO_2 from each experimental treatment over the 7-day study

N_2O (nmol g^{-1} DW)					
**Day**	**Control**	**MIX**	**SMX**	**DAN**	**ETM**
**2**	0.01 ± 0.001	0.01± 0.001	0.01 ± 0.001	0.01 ± 0.002	0.01 ± 0.02
**4**	0.01 ± 0.0004	0.01 ± 0.001	0.01 ± 0.0002	0.01 ± 0.001	0.01 ± 0.001
**7**	0.01 ± 0.0003	0.01 ± 0.0001	0.01 ± 0.0003	0.01 ± 0.0003	0.01 ± 0.0001

N_2 (μmol g^{-1} DW)					
**Day**	**Control**	**MIX**	**SMX**	**DAN**	**ETM**
**2**	17.5 ± 1.04	17.5 ± 0.60	17.5 ± 0.66	20.0 ± 1.0	16.8 ± 1.71
**4**	16.9 ± 0.92	17.0 ± 0.90	24.8 ± 1.41	20.1 ± 0.27	20.1 ± 0.63
**7**	17.2 ± 0.4	18.9 ± 0.69	16.2 ± 0.46	16.2 ± 0.66	17.5 ± 0.72

CH_4 (nmol g^{-1} DW)					
**Day**	**Control**	**MIX**	**SMX**	**DAN**	**ETM**
**2**	4.8 ± 0.5	16.1 ± 4.5	10.4 ± 2.3	11.4 ± 2.04	7.9 ± 2.0
**4**	13.6 ± 1.5	22.5 ± 2.5	20.1 ± 3.8	11.5 ± 1.5	8.9 ± 1.2
**7**	36.0 ± 4.0	32.6 ± 1.8	29.4 ± 2.2	41.7 ± 7.8	43.5 ± 6.7

CO_2 (nmol g^{-1} DW)					
**Day**	**Control**	**MIX**	**SMX**	**DAN**	**ETM**
**2**	592 ± 60.9	359 ± 17.4	322 ± 14.1	575 ± 89.2	550 ± 65.3
**4**	557 ± 94.6	779 ± 27.7	659 ± 25.0	680 ± 48.9	672 ± 39.2
**7**	685 ± 55.4	725 ± 16.0	689 ± 45.5	814 ± 62.1	757 ± 39.2
