# Supplementary material for: Are nitrogen and carbon cycle processes impacted by common stream antibiotics? A comparative assessment of single vs. mixture exposures
Source: PLoS One. 2022 Jan 5;17(1):e0261714. doi: 10.1371/journal.pone.0261714 (PMC8730405; doi:10.1371/journal.pone.0261714)
Supplement: S5 File — Mean gene copy number per gram of sediment for each functional gene investigated in the study. (PDF) [file pone.0261714.s005.pdf]

S5

Mean gene copy number per gram of sediment for each functional gene investigated in the study

	**Genes**			
	mcrA	***nosZ***	***nirK***	***amoA***
**Control**	5.25E+05	8.21E+05	1.93E+05	1.01E+06
**SMX**	6.87E+05	7.93E+05	1.86E+05	1.09E+06
**DAN**	6.04E+05	8.17E+05	2.46E+05	8.22E+05
**ETM**	4.80E+05	7.88E+05	1.86E+05	9.16E+05
**MIX**	5.38E+05	8.05E+05	2.33E+05	1.56E+06
